# Genome wide association study of plant height and tiller number in hulless barley

**DOI:** 10.1371/journal.pone.0260723

**Published:** 2021-12-02

**Authors:** Yixiong Bai, Xiaohong Zhao, Xiaohua Yao, Youhua Yao, Likun An, Xin Li, Yong Wang, Xin Gao, Yatao Jia, Lulu Guan, Man Li, Kunlun Wu, Zhonghua Wang

**Affiliations:** 1 State Key Laboratory of Crop Stress Biology for Arid Areas, College of Agronomy, Northwest A&F University, Yangling, Shaanxi, China; 2 Qinghai University, Qinghai Academy of Agricultural and Forestry Sciences, Qinghai Key Laboratory of Hulless Barley Genetics and Breeding, Xining, Qinghai Province, China; 3 Good Agricultural Practices Research Center of Traditional, Chongqing Institute of Medicinal Plant Cultivation, Chongqing, China; Institute of Genetics and Developmental Biology Chinese Academy of Sciences, CHINA

## Abstract

Hulless barley (*Hordeum vulgare* L. var. *nudum*), also called naked barley, is a unique variety of cultivated barley. The genome-wide specific length amplified fragment sequencing (SLAF-seq) method is a rapid deep sequencing technology that is used for the selection and identification of genetic loci or markers. In this study, we collected 300 hulless barley accessions and used the SLAF-seq method to identify candidate genes involved in plant height (PH) and tiller number (TN). We obtained a total of 1407 M paired-end reads, and 228,227 SLAF tags were developed. After filtering using an integrity threshold of >0.8 and a minor allele frequency of >0.05, 14,504,892 single-nucleotide polymorphisms (SNP) loci were screened out. The remaining SNPs were used for the construction of a neighbour-joining phylogenetic tree, and the three subcluster members showed no obvious differentiation among regional varieties. We used a genome wide association study approach to identify 1006 and 113 SNPs associated with TN and PH, respectively. Based on best linear unbiased predictors (BLUP), 41 and 29 SNPs associated with TN and PH, respectively. Thus, several of genes, including *Hd3a* and *CKX5*, may be useful candidates for the future genetic breeding of hulless barley. Taken together, our results provide insight into the molecular mechanisms controlling barley architecture, which is important for breeding and yield.

## Introduction

*Hulless barley* (*Hordeum vulgare* L. var. *nudum*) is a variety of cultivated barley that is also known as naked barley because the separation of its grains and glumes creates a ‘naked’ caryopsis [1]. Hulless barley is mainly cultivated on the Qinghai–Tibet Plateau [2], because it possesses key adaptations to extreme environments. In total, approximately 356,000 ha are occupied by hulless barley cultivation in China. Barley is a staple of the Tibetan diet and confers significant nutritional and health benefits. It is also widely used in the winemaking and food processing industries, and barley seedlings and straw are also used as high-quality forage [3] and play a vital role in animal husbandry on the Tibetan plateau. Therefore, the breeding of high-yield hulless barley varieties is desirable for the future development of the hulless barley industry.

Plant architecture strongly affects light capture [4], and the distribution of nutrients between the vegetative and reproductive organs [5] indirectly affects crop production. The height of the main stalk and the formation of tillers are major architectural components of cereal plants [6]. The orientation and height of the stalk and tillers affect the cover and spatial distribution of the cereal plant. Plant height (PH) is mainly controlled by members of the *Rht* gene family [7–10], which regulate gibberellin biosynthesis and signal transduction in many crops [11,12]. In contrast, tiller number (TN) is regulated by a complicated gene network. In rice, overexpression of *OsMADS57* resulted in increased tiller outgrowth relative to wild-type plants, suggesting that *OsMADS57* plays a key role in rice tillering [13]. In addition, *OsMIR444a*, which regulates *OsMADS57*, together with *OsTB1*, was found to target *D14*, to control tillering [14]. Moreover, *MOC1* was also characterised as a key regulator involved in the control of rice tillering and branching [15]. Another study of wild rice showed that the *PROG1* gene controls aspects of both the tiller angle and the number of tillers [16]. In wheat, the *tin3* gene was localized to the long arm of chromosome 3A^m^; this gene differed from the wild-type counterpart by a single recessive mutation and reduced the number of tillers produced by the plant [17]. Another study of wheat found that tillering was related to lignin and cellulose metabolism, cell division, cell cycle processes, and glycerophospholipid metabolism and that modulation of GRAS, GRF, and REV the transcription factor families might decrease tillering [18]. Taken together, marker-based studies of many crops have identified numerous quantitative trait loci (QTLs) that are closely linked to tillering [19–21]. Relative to other crops, hulless barley is exposed to lower temperatures and higher winds, which renders the stem thinner and softer and can cause lodging. However, the broad-sense heritability of PH and TN in natural populations of hulless barley remains unknown.

Genome wide association studies (GWASs) are conducted via population genotyping using high-throughput sequencing data. In these studies, different models are used to associate objective traits with markers [22,23]. The most suitable populations for this type of analysis are natural populations with different genetic bases, rather than cross-derived segregating populations. GWAS data should be collected from multiple environments and multiple years to maximise robustness. Compared with traditional QTL analyses, GWAS can map QTLs more efficiently and identify genes responsible for multiple agronomic traits with greater ease [24]. GWASs have proven to be a useful method to identify genomic regions associated with complicated quantitative traits, such as drought resistance [25], floret fertility [26], malting quality [27], agronomic traits [28], lodging traits [29], disease resistance [30], and seed vigour [31]. For example, a previous study of soybean used a GWAS to identify *Dt1* and a pectin lyase-like gene as stably associated with PH [32]. In wheat, two stable SNPs, Excalibur_c11045_236-A and BobWhite_c8436_391-Tas, were identified for the development of cleaved amplified polymorphic sequence markers associated with TN in natural populations; the presence of these SNPs increased the rate of tillering by 14.78% and 8.47% [33], respectively. In barley, an association analysis identified three sugar-related QTLs affecting TN on chromosomes 3H, 4H and, 5HS, which encompass *HvHXK9* and *HvHXK6*, *HvSUT1* and, *HvSUT2*, respectively. Ten significant chromosomal regions affecting PH were identified. Among them, the strongest associations with PH were as follows: on 4H, between 59.6 and 59.8 cM, co-located with *HvD4*; and on 1H, between 10.9 and 13.4 cM, a region lacking known candidate genes [34]. Despite these results, few studies have identified QTLs related to PH and TN in hulless barley, and those reports in which the authors have used genome wide association analyses to identify regions associated with plant architecture have not provided candidates that are known to be present in hulless barley. Thus, the genetic basis of PH and TN in hulless barley remains unclear, which restricts the use of marker-assisted breeding in this crop. Here, we aimed to associate plant architecture traits with genetic variation in natural populations of hulless barley and to develop new SNP markers that are closely linked to PH and TN.

The article aimed to identify the plant architecture distribution and genetic variation of natural populations of in hulless barley, and to associate new SNP markers closely linked to the PH and TN traits. Our results shed light on understanding of the genetic basis of plant architecture, provided QTLs and markers that can be used by breeders, and constructed a theoretical basis for fine mapping and for marker-assistance selecting breeding.

## Materials and methods

### Plant materials

A natural association population that included 300 accessions of hulless barley was used as the source plant material for this study. This natural population was sourced worldwide, although many accessions came from China. Each representative accession was self-pollinated in the spring of 2016, and the leaves of plant seedlings were sampled to extract genomic DNA for SLAF analysis.

### Experimental design and trait measurements

Tests were conducted at three experimental farms: one was located at the Qinghai Academy of Agriculture and Forestry Sciences (named XN, 36.62°N,101.77°E), and another one was located at the Haibei Institute of Agricultural Sciences (named HB, 37.02°N, 100.55°E), and the third one was located at Guinan (named GN, 35.82°N, 101.12°E), All in Qinghai Province, China. Hulless barley accessions were grown using a randomised block design complete with three replicates each in four growing periods from April 2016 to August 2019 (referred to henceforth as 16–19, in total eight environments). At maturity, 10 representative plants were selected for measurements. The PH and productive TN of each plant were then assessed. PH was measured as the height from the base of the stem to the tip of the main inflorescence. At the harvest stage, TN was measured as the number of branches on the main shoot. The mean value of these 10 plants was used to represent the trait value of an accession. Analysis of variance and correlations among phenotypic traits were conducted using IBM SPSS version 20.0 (IBM, Chicago, USA).

### SLAF library construction and sequencing

DNA was extracted from the leaves of hulless barley plants using the CTAB method. The barley genome was used as a reference for restriction digestion prediction (ftp://ftp.ensemblgenomes.org/pub/release-36/plants/fasta/hordeum_vulgare/dna/) [35], and RsaI and EcoRV-HF (New England Biolabs, NEB) were selected to digest the hulless barley genome. SLAF tags (364–414 bp) were then collected and linked to dual-index sequencing adapters to construct the SLAF library [36]. Paired-end sequencing was conducted on selected SLAFs using high-throughput sequencing platform (Illumina HiSeq:Illumina, Inc; San Diego, CA, USA).

### *In silico* mapping of SNPs

After filtering out low-quality reads and adapter sequences, the remaining high-quality reads were aligned to the *Hordeum vulgare* v2 reference genome using the BWA software [35]. SNPs were then detected using GATK 3.8 and SAMtools 1.9. The group of SNPs that were detected using both methods was designated as the final group of SNPs and was retained for further analysis. An integrity threshold of >0.8 and a minor allele frequency (MAF) > 0.05 were used to call SNPs with high consistency in the sequencing population.

### Phylogenetic analysis

A phylogenetic tree of the sample sequences was constructed using the neighbour-joining algorithm implemented in MEGA6 [37]. A principal component analysis (PCA) was carried out using the EIGENSOFT software. Relative kinship was estimated using SPAGeDi [38]. Decay of linkage disequilibrium (LD) was evaluated, as was the distance between sites in base pairs (bp), using non-linear regression, as implemented in the R package.

### Genome-wide association analysis

Best linear unbiased predictors (BLUP) were estimated for each environment for each trait based on a mixed linear model using the lme4 package. The BLUP values and single year and location values for each genotype were used for the association analysis. All filtered SNPs from the 300 accessions were used for GWAS. A GWAS for all traits (based on LM,LMM, FaST-LMM, and EMMAX models) was conducted using the GEMMA (https://bioinformaticshome.com/tools/gwas/descriptions/GEMMA.html), FaST-LMM (https://www.microsoft.com/en-us/download/confirmation.aspx?id=52588), and EMMAX (http://csg.sph.umich.edu//kang/emmax/download/index.html) software, with default settings used in each step.

## Results

### Phenotypic analysis of PH and TN

**Fig 1:**
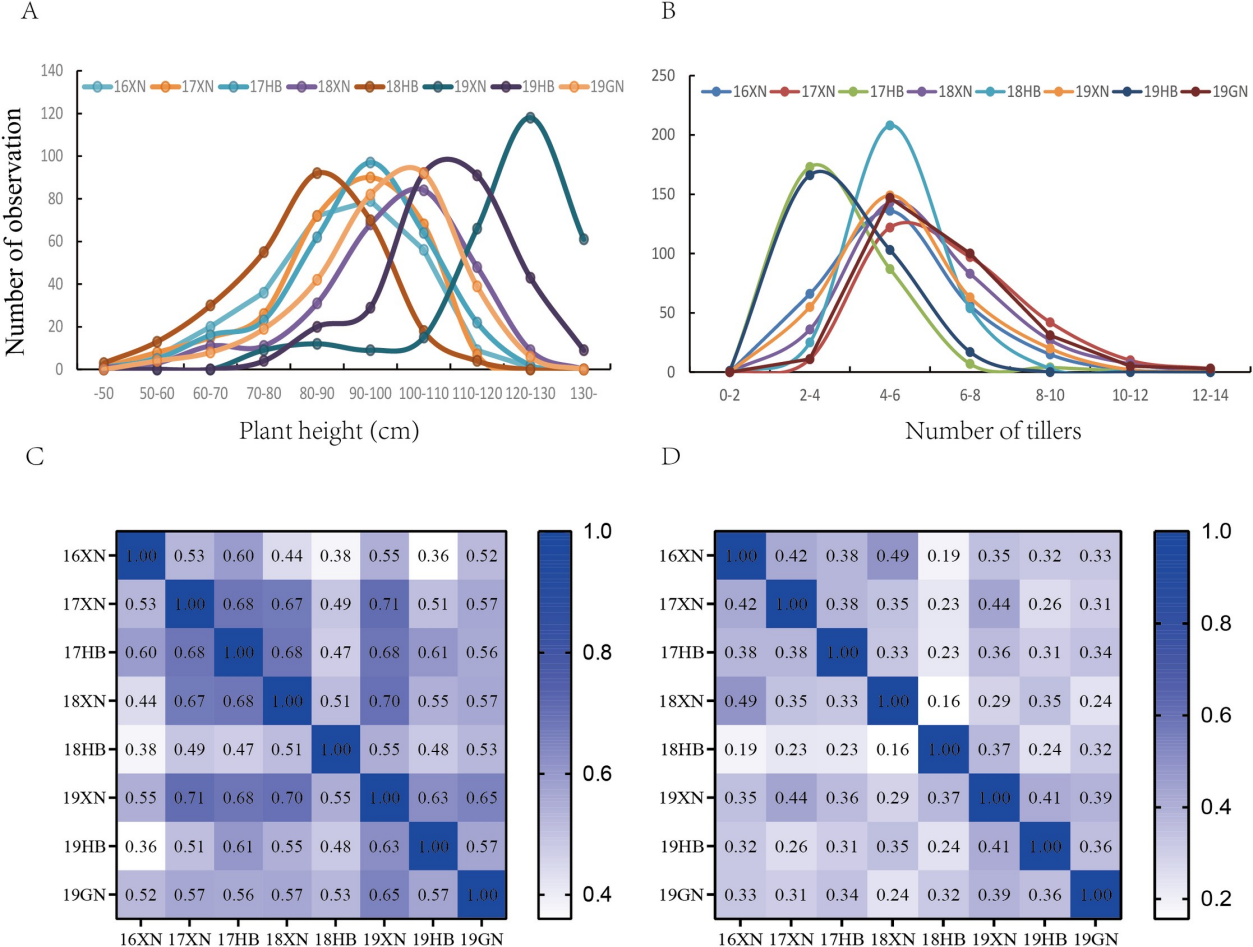
Distribution and correlation of PH and TN in hulless barley germplasms. The distribution of PH (A) and TN (B) at years and locations. The correlation matrix of PH (C) and TN (D) at years and locations. All the significance were P <0.01. 17–19, mean 2017–2019 years.

The distribution of PH and TN was skewed and leptokurtic (Fig 1A and 1B, S1 Table). The comparison of the data of different locations and years, revealed a PH ranging from 55.38 to 127.1 at XN in 2016, 47.25 to 118.8 at XN in 2017, 43.25 to 122.5 at HB in 2017, 49.93 to 127.9 at XN in 2018, 40.25 to 113.8 at HB in 2018, 73.78 to 154 at XN in 2019, 70.70 to 140.6 at HB and 56.38 to 129 at GN in 2019. In turn, the TN ranged from 2.63 to 11 at XN in 2016, 2.75 to 16.3 at XN in 2017, 1.75 to 11 at HB in 2017, 2.75 to 12.5 at XN in 2018, 2.33 to 10.5 at HB in 2018, 2 to 12.5 at XN in 2019, and 1.75 to 7.5 at HB and 3 to 13 at GN in 2019.

PH and TN were significantly correlated across the three locations and 4 years, with a correlation coefficient of 0.483–0.705 and 0.156–0.44, respectively (Fig 1C and 1D). The broad-sense heritability (H^2^) values for PH and TN were 80.66% and 78.92%, respectively (Table 1), suggesting that both traits are stably inherited. Further analysis of the interaction effects of year, location and genotype, revealed that three factors were significantly correlated to PH and TN; moreover, we found significant interaction effects of L×Y, L×G, L×Y×G (S2 Table), suggesting that the PH and TN traits are modulated by a combination of genetic and non-genetic factors.

**Table 1:** Variance components and broad-sense heritability for PH and TN in the hulless barley population.

Trait	Variance component^a^			Residuals	H^2^ (%)^b^
	G	E	G×E		
PH	85.29	40.76	35.08	82.37	80.66
TN	2.36	0.24	0.65	4.02	78.92

^a^ G and E indicate genotype and environment, respectively, and G × E indicate interaction of G and E.

^b^ Family mean-based broad-sense heritability.

### Construction of a genomic library and identification of SNP markers

**Fig 2:**
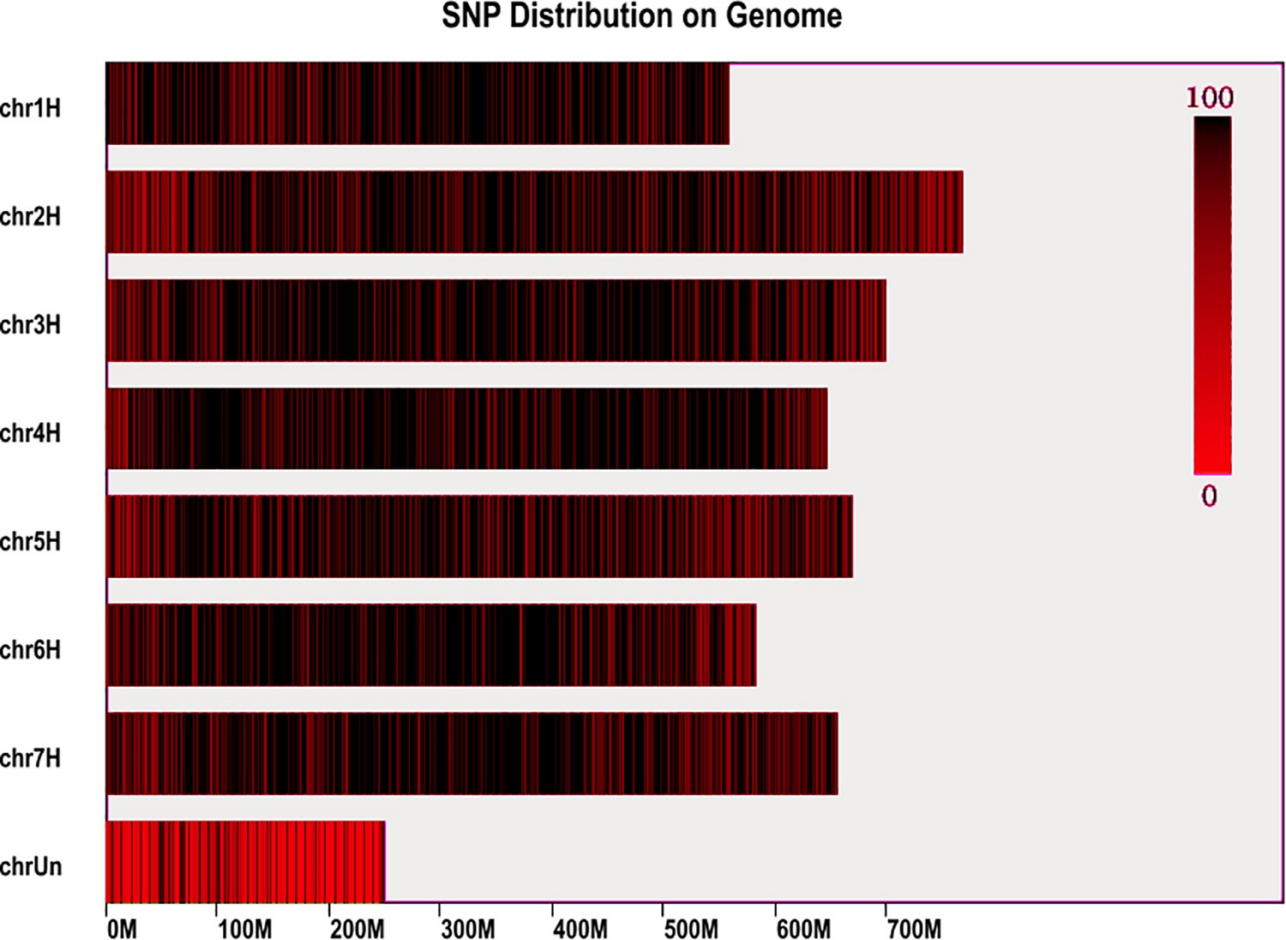
The distribution of SNP locus on the chromosome. The color bar showed the number of SNP.

Next, we constructed a genomic library for hulless barley and used the rice genome as a control. According to prediction, the length of the SLAF tags ranged from 364 to 414 bp, with 228,227 SLAF tags obtained in total. Moreover, we found that the SLAF tags were evenly distributed throughout the genome (S3 Table, Fig 2). In total, we obtained 1407 M paired-end reads from the 300 hulless barley accessions. The average number of reads obtained for each sample was 4.7 M, and the average Q30 and GC content values were 94.2% and 43.5%, respectively. These results indicated that our sequencing results could be used for further analyses (S4 Table). The efficiency of double-ended alignment to a reference genome was 90.60% of the control alignment to the rice genome. The efficiency of enzyme digestion in the control was 95.59%, and the distribution of fragments showed that the digestion reaction proceeded normally (S1 Fig).

In this study, the average sequencing depth of the sample was 13.53×. In addition, 379,010 SLAF labels and 273,654 polymorphic labels were identified (Tables 2 and S5). From the intersection of GATK and SAMtools, a total of 14,504,892 SNPs were obtained. After filtering using an integrity threshold of >0.5 and a MAF of >0.05, a total of 560,704 SNPs were obtained (S6 Table).

**Table 2:** SLAF label and polymorphism SLAF label chromosome distribution statistics.

Chromosome ID	SLAF number	Polymorphic SLAF
chr1H	46,076	33,393
chr2H	59,310	42,834
chr3H	57,371	42,438
chr4H	53,150	39,227
chr5H	52,342	37,868
chr6H	47,376	35,439
chr7H	52,088	38,605
chrUn	11,297	3,850
Total	379,010	273,654

### Evolutionary analysis

**Fig 3:**
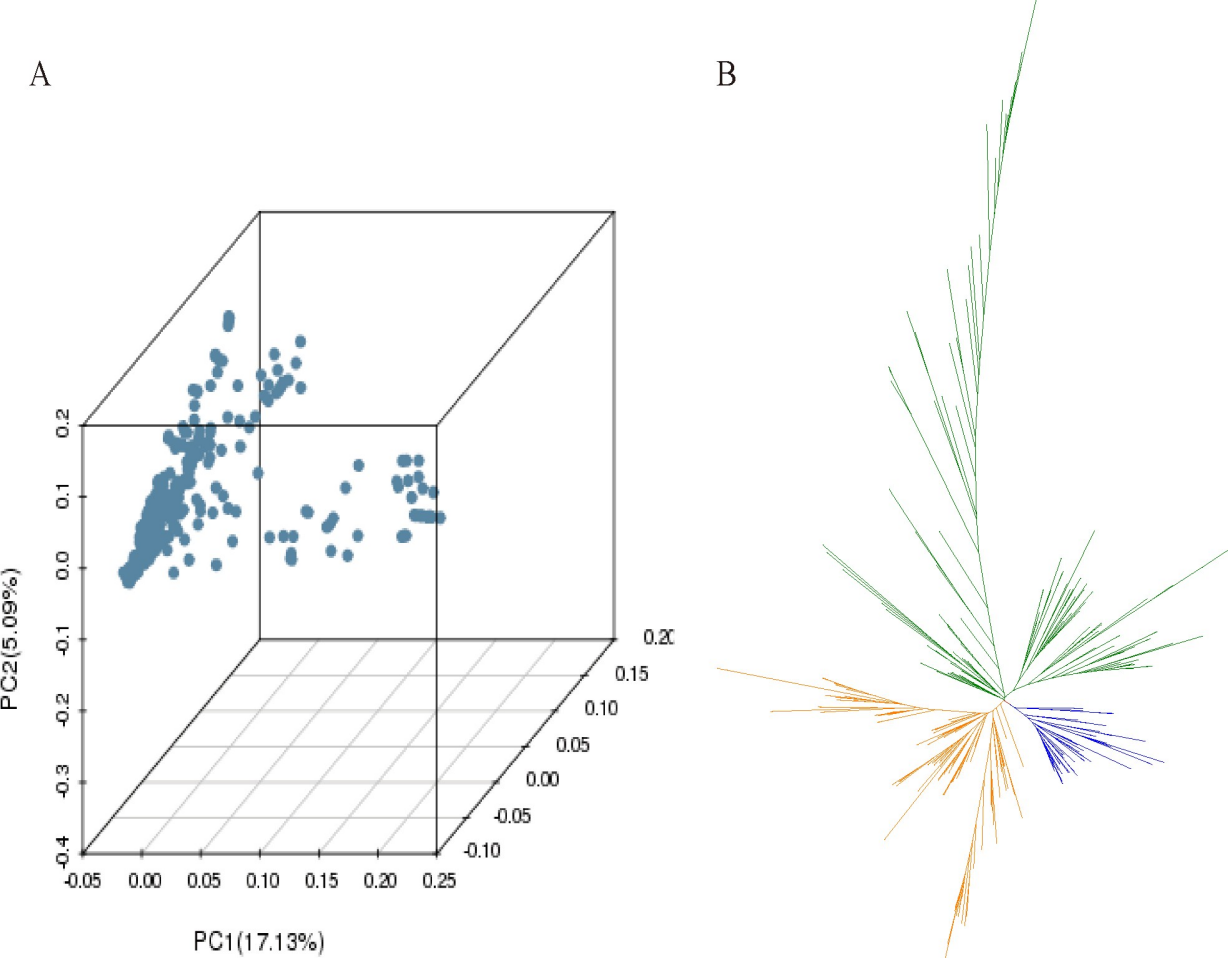
The phylogenetic evolution tree and principal component of the all accessions. (A) Principal component analysis of the 300 accessions. (B) The phylogenetic evolution tree of the 300 sequencing accessions.

Based on the SNP dataset, we constructed a phylogenetic tree of the 300 accessions. However, based on the PCA plot, the two subpopulations (landraces and varieties) could not be clearly segregated (Fig 3A), likely because a large proportion of the cultivated varieties were derived from Qingke barley landraces. From the phylogenetic tree, we found that although the 300 accessions could be divided into three main branches, the local varieties could not be strictly differentiated within each branch (Fig 3B). These results were similar to those reported by Li et al. [39].

### Association mapping

**Fig 4:**
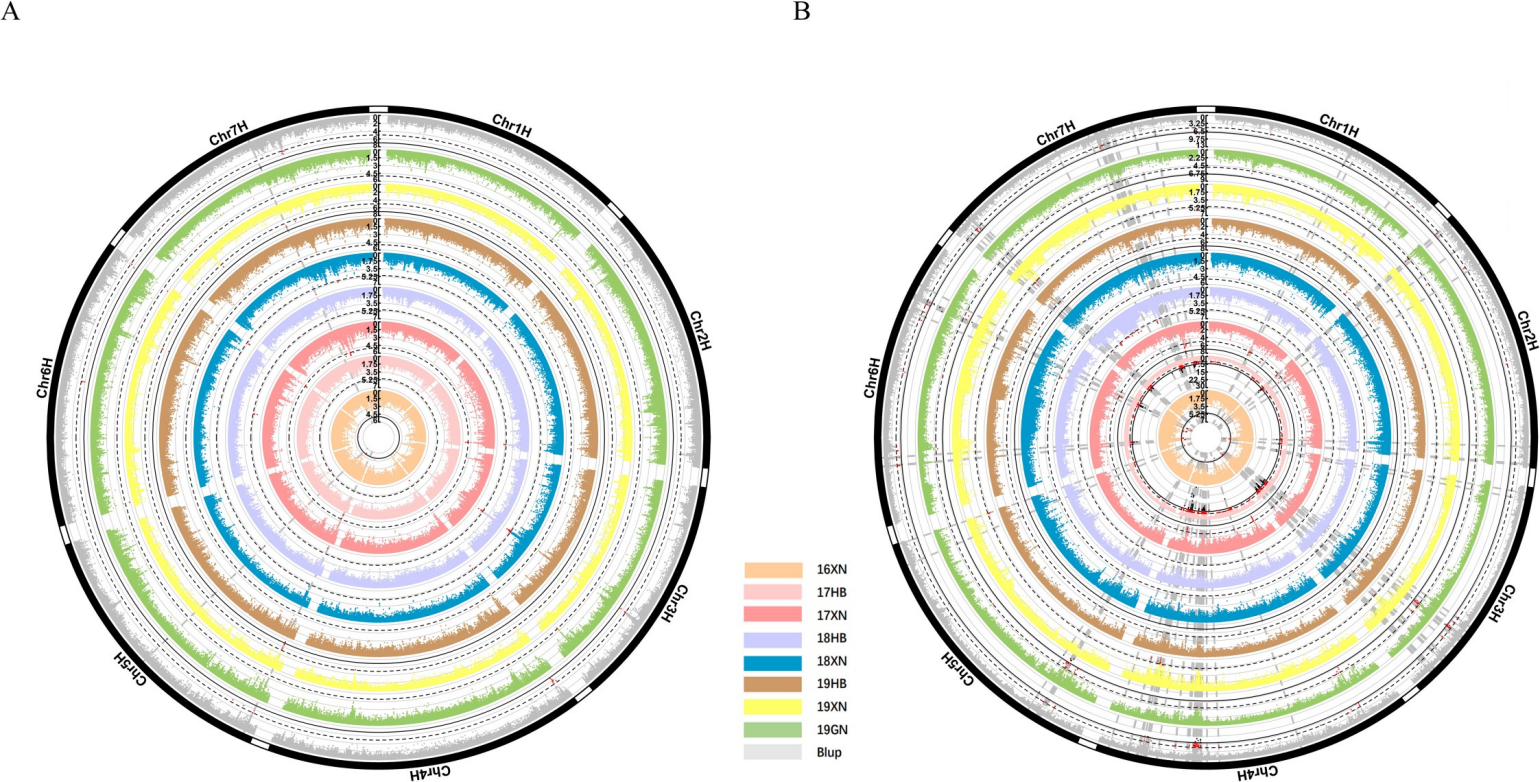
Manhattan plots for genome-wide association mapping of the PH (A) and Tiller (B) by using an LMM method. Negative log10-transformed P values from a genome-wide scan are plotted against position on each of 7 chromosomes. Red and Black dots indicates the genome-wide significance threshold (P> = 5 and 7, respectively).

Association mapping was conducted in GEMMA using LM, LMM, fastlmm and, emmax for all SNP markers. The BLUP values and single environment values for each genotype were used in the association analysis. The results of LMM showed that there were 113 SNP loci related to PH across eight environments (Fig 4A, S7 Table) and that these loci were distributed on seven different chromosomes. We also found 1006 SNP markers related to TN across eight environments using LMM, and these SNP were distributed on seven chromosomes (Fig 4B, S8 Table). Based on the results of the association analysis of BLUP values, we screened out 41 and 29 SNP loci related to PH and TN, respectively. Further analysis of the coding genes located within 100 kb upstream and downstream of these loci revealed that 11 of these 70 SNP markers were located at sites with no nearby coding genes, whereas the remaining 59 sites were located near one or more coding genes. Of these, we found 62 and 29 coding genes locted within 100 kb upstream and downstream of the PH and TN SNP loci, respectively. These SNPs were located on multiple chromosomes, indicating that the TN and PH traits of hulless barley are controlled by multiple genes.

The results of the evaluation of the decay of LD showed that the predicted value of *r*^*2*^ declined to 0.1 within 1 M (S2 Fig). In this study, we chose 1 M as the LD decay distance, with the interval of 50 kb upstream and downstream of the peak SNP (2 M in total) being defined as a QTL locus. In total, we identified 24 QTLs distributed across the barley genome related to the PH and TN traits (S9 Table). The annotation showed that one SNP region was intronic, one was exonic, four were located upstream, and the others were intergenic. For example, the chr1H_394787146 SNP locus was located in exon regions of the HORVU1Hr1G053420 gene, with the nucleotide at this position being changed from T to C, causing an amino acid change from threonine (T) to alanine (A) (S3 Fig).

### Identification of candidate genes

Next, we performed a BLAST annotation of all coding gene sets within the above-mentioned SNP loci (S9 Table). We found that HORVU2Hr1G004610 had a cytochrome P450 homology, and was involved in reduced PH in rice [40–42]. Two F-box family proteins (HORVU1Hr1G048700, and HORVU4Hr1G080860) and an E3 ubiquitin-protein ligase (HORVU4Hr1G080840) homologs may induce shorter plants [43,44]. These results indicated that the *Hd3a* (HORVU2Hr1G072750) gene, which is associated with the chr2H_523339528 locus, is closely related to TN [45]. The homolog of cytokinin dehydrogenase 5 (CKX5, HORVU3Hr1G075920) was involved in the strigolactone signalling pathway in rice and *Arabidopsis thaliana* [43,44,46]. Moreover, the HORVU1Hr1G053990 gene was homolog of *NRT1*, which modulates shoot architecture in *Arabidopsis thaliana* [47].

**Fig 5:**
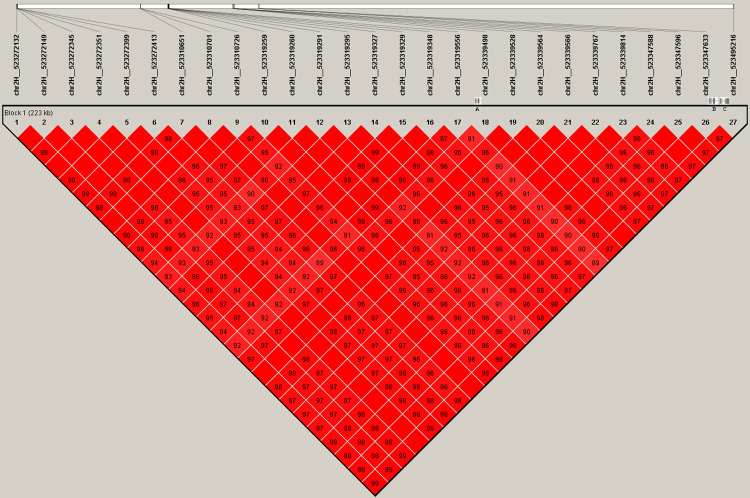
The candidate range associated with tiller number in hulless barley. A region of 230kb are indicated in middle panel (From left to right: HORVU2Hr1G072730, HORVU2Hr1G072740 and HORVU2Hr1G072750 (Hd3a)). Box color mean: White (LOD < 2, D’ < 1), blue (LOD < 2, D’ = 1), shade of pink (LOD≥2, D’ < 1) and bright red (LOD≥2, D’ = 1).

Among these SNPs, two SNPs (chr3H_567116810 with a P-value of 1.55E-11 and chr3H_567112423 with a P-value of 7.62E-9) were significantly associated with the TN of hulless barley. The candidate genes associated with the lead SNP included CKX5. For the Hd3a-related SNP chr2H_523339528, we analysed the haploblock with 230 k from SNP chr2H__523272132 to chr2H__523495216. The haploblock contained three genes, i.e., HORVU2Hr1G072730 (antisense strand of chr2H:523338401–523339409), HORVU2Hr1G072740 (chr2H:523358478–523359486) and HORVU2Hr1G072750 (Hd3a, chr2H:523377399–523379178) (Fig 5). The lead SNP (chr2H_523339528) is located 119 bp, 19 kb and 40 kb upstream of HORVU2Hr1G072730, HORVU2Hr1G072740 and HORVU2Hr1G072750, respectively, and may be located in the promoter region of these genes.

## Discussion

SLAF-seq is a high-throughput sequencing-based genotyping technology that is used to identify large numbers of SNPs and develop biomarkers. Hulless barley is the most important cereal crop grown on the Qinghai–Tibet Plateau, as it has been for approximately 3,500 years, and is used for wine production and consumption [39]. Hulless barley is an ancient crop that is mainly distributed throughout high-altitude and economically poor areas in the Chinese provinces of Tibet, Qinghai, Sichuan, and Yunnan [48]. However, to date, research on the genetic basis of key traits of hulless barley remains underdeveloped. Moreover, this lack of knowledge restricts the application of modern breeding techniques to hulless barley and has hampered the improvement of the yield and quality of this crop through molecular breeding.

In a recent study, Li *et al*. collected 308 hulless barley accessions, including 206 Qingke landraces, 72 Qingke varieties, and 30 varieties, and planted them together in Tibet to identify genetic loci associated with heading date, PH and, spike length using a GWAS-based framework. Those authors identified 62 QTLs associated with these three important traits and mapped 114 known genes related to vernalization and photoperiod, among others [39]. Using an LD decay analysis, Li *et al*. found that the r^2^ remained >0.1 for over 80 Mb; however, in our study, this value was about 1 Mb; whether this discrepancy is related to the variety of the materials used in the two studies remains to be further studied. Previously, Dai et al. found significant genetic differentiation between wild barley accessions from the Near East and Tibet and used transcriptome profiling of cultivated and wild barley genotypes to reveal the multiple origins of domesticated barley [48,49]. In our study, we focused mainly on traits related to plant architecture, such as PH and TN. These traits are closely related to lodging resistance and the mechanised harvesting of barley [29,50].

In rice, previous studies have shown that the *DWARF3* (*D3*), *D10*, *D14*, *D17*, *D27*, and *D53* genes are involved in strigolactone biosynthesis and perception. This is the main pathway that controls TN in rice [43,44,51–58]. Similar results were found obtained for spring barley [34]. In this study, we observed that TN was associated with multiple genes involved in strigolactone biosynthesis and perception, such as *Hd3a*, ubiquitin-protein ligase and *CKX5*. As mentioned above, *Hd3a* is a homolog of the *FT* gene or TFL1 protein, which is involved in flowering and accumulates in axillary meristems to promote branching [45,59]. *CKX5* is a homolog of *OsCKX9*, the mutants and overexpression transgenic plants of which yielded significant increases in tiller number and decreases in plant height [46]. In addition, *NRT1* has also been reported to be closely related to tiller and plant architecture development [47]. The identification of these marker genes indicates that the screening results have high reliability. Rice and hulless barley are similar species (family *Poaceae*) and may have similar regulatory networks, which would explain why we found that the same SNP loci were linked to TN in hulless barley.

Previous studies have shown that QTLs located on chromosomes 1H, 2H, 5H, and 7H were significantly associated with PH [34,39]. In spring barley, chromosomes 1H (95.9–96.9 cM), 2H (6.5–8.9 cM), 4H (44.9 cM) and 5H (143.7–146.1 cM), have also been linked to increased productive tillering [34]. Previous studies have found SNP loci adjacent to regions containing candidate genes such as *BRASSINOSTEROID-6-OXIDASE* (*HvBRD*) [60] and *HvDRM1* [61]. In this study, these genes were not screened out in our results; however, some candidate genes, such as OsNAP1, function in cell proliferation and cell expansion, which may be closely related to PH [62]. In this study, HORVU1Hr1G053420 is the only gene that had SNP loci in exons and caused an amino acid change. However, we did not find a report about the function of this gene or its homology.

Similar to previous studies, we also found that genes linked to PH were located on different chromosomes, and that their activity may depend on the environment and/or experimental treatment [63,64]. Li et al. planted all 308 barley accessions in three locations in Tibet: Lhasa (N29°36′, E91°06′), Namling (N29°18′, E88°46′), and Nyingchi (N29°39′, E94°21′). However, in our study, all 300 accessions were planted at the Qinghai Academy of Agriculture and Forestry Sciences (N36°62′, E 101°77′) and at the Haibei Institute of Agricultural Sciences (N 37°02′, E 100°55′). The interaction effects showed two phenotype caused by the combination of genetic and non-genetic factors. Moreover, the GWAS results revealed only a small number of common SNP loci in multiple environments. Our study showed that SNPs related to PH and TN were located on multiple chromosomes; however, differences between the candidate SNPs and associated genes reported here and those reported in previous studies may reflect the effect of different environments [39].

## Conclusions

In this study, we identified SNP loci associated with PH and TN in hulless barley using SALF methods via high throughput sequencing technology. In total, 560,704 screened SNP markers were used for GWAS analysis, and 1006 and 113 SNP loci were related to TN and PH, respectively. Moreover, our results showed that PH and TN were affected by the combination of genetic and non-genetic factors. Based on the BLUP results, 41 and 29 SNP loci related to PH and TN were screened out, respectively. Analysis of these target genes in 100 kb windows upstream and downstream of the SNPs associated with PH and TN,led to the screening out of 91 target genes. The candidate genes included *HvHd3a*, *HvCKX5*, cytochrome P450, F-box, etc. However, further research is needed to elucidate how these candidate genes are expressed in hulless barley and to clarify their roles in the control of PH and TN. These findings may be relevant for the search for molecular markers linked to key agronomic traits in highland barley and may be useful for future marker-assisted breeding programmes of this important crop.

## Supporting information

S1 Fig The distribution of observed control insert size. (PNG)

S2 Fig The linkage disequilibrium decay. (PDF)

S3 Fig The SNP loci chr1H_394787146 in HORVU1Hr1G053420. (PDF)

S1 Table The raw data of PH and TN. The 17–19 in line 1 means year 2017–2019.
(XLS)

S2 Table Interaction effects of year, location and genotype. (XLSX)

S3 Table Number of prediction SLAF tags on each chromosome. (XLSX)

S4 Table Sequencing data statistics for each sample. (XLSX)

S5 Table The number of SLAF tags. (XLSX)

S6 Table The statistics of sample SNP. (XLSX)

S7 Table The list of SNP makers about PH. (CSV)

S8 Table The list of SNP makers about TN. (CSV)

S9 Table The annotation of candidate genes. (XLSX)
