# Supplementary figures and images for: Genome wide association study of plant height and tiller number in hulless barley
Source: PLoS One. 2021 Dec 2;16(12):e0260723. doi: 10.1371/journal.pone.0260723 (PMC8639095; doi:10.1371/journal.pone.0260723)

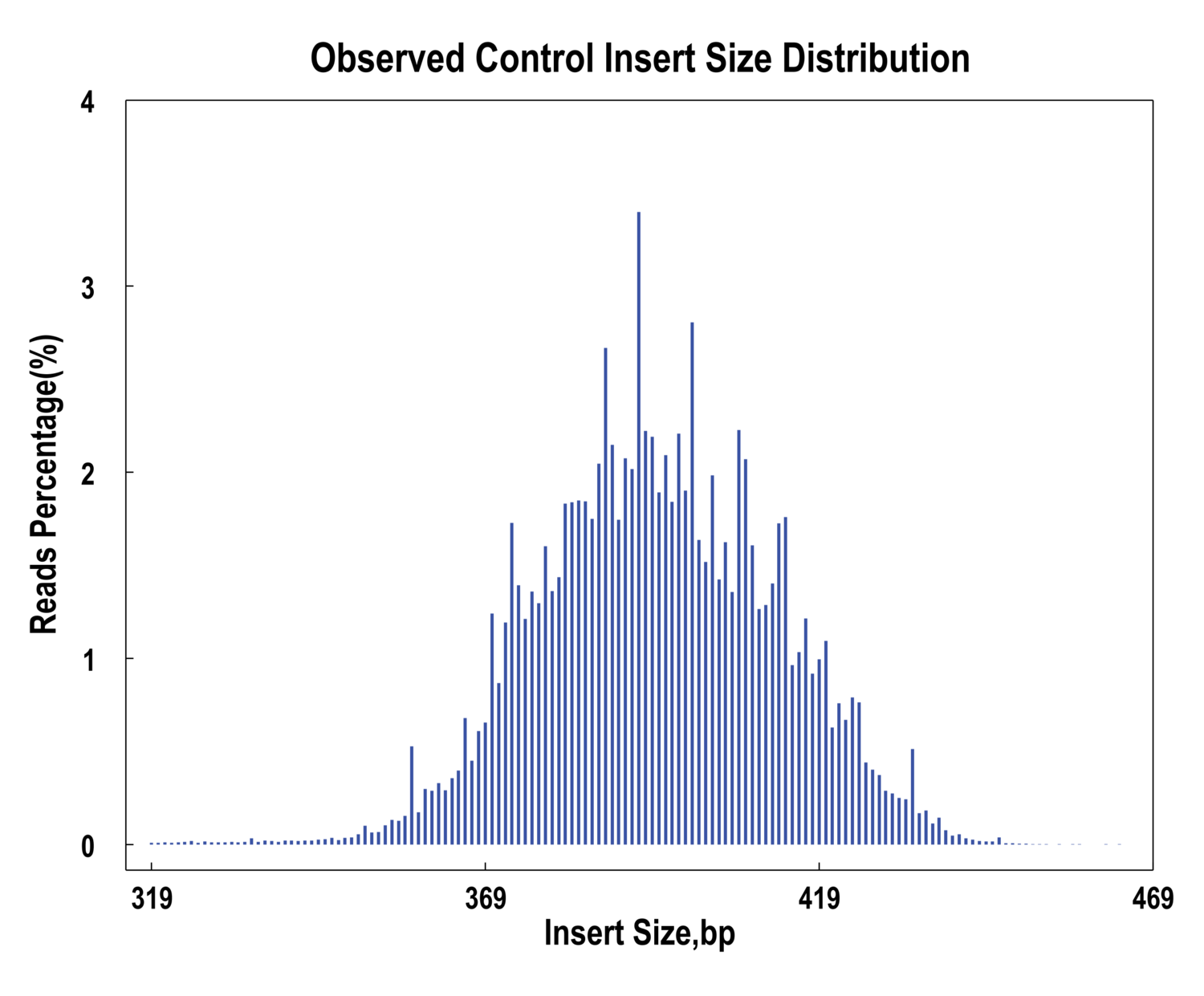

Supplement: S1 Fig — (PNG) [file pone.0260723.s001.png]

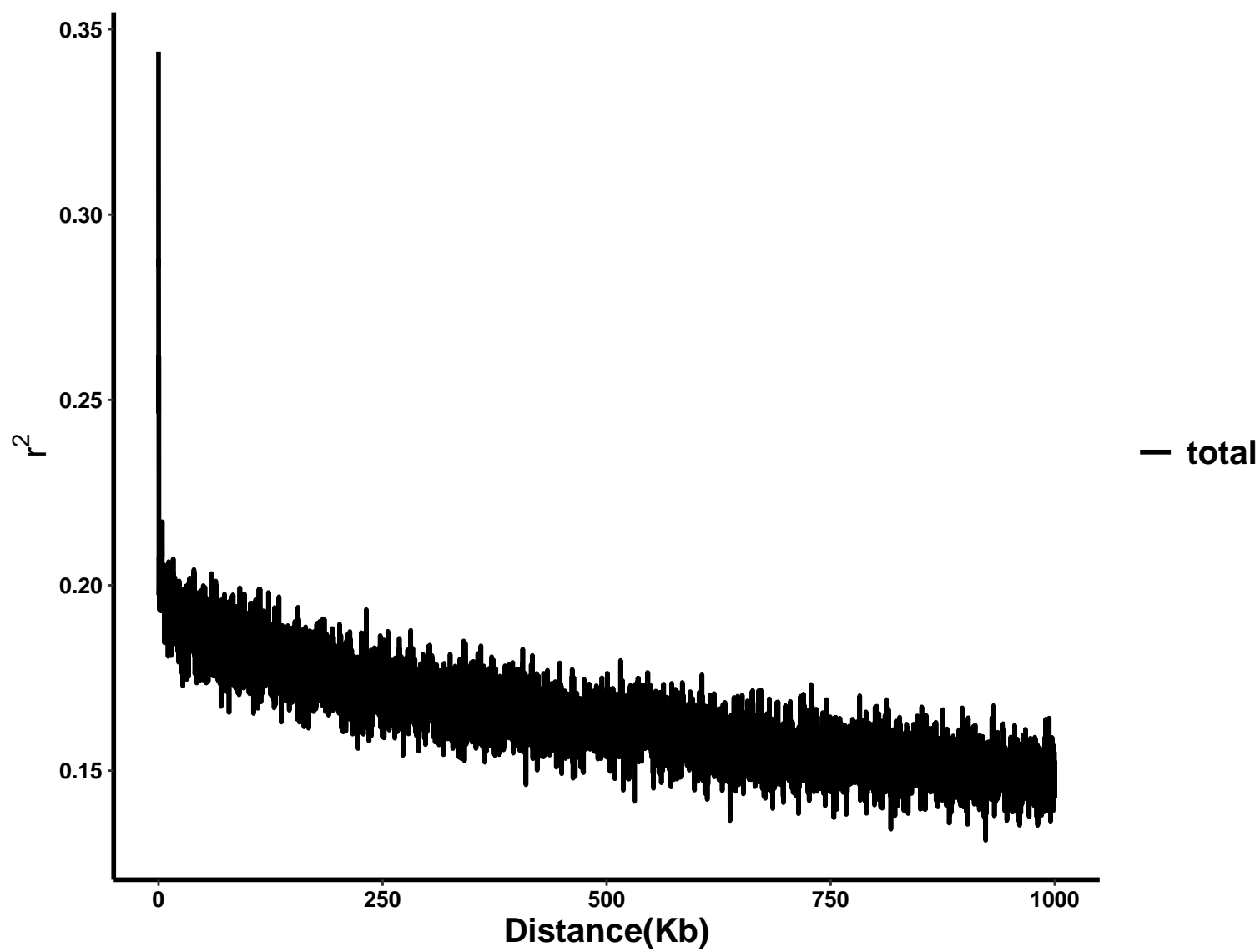

Supplement: S2 Fig — (PDF) [file pone.0260723.s002.pdf]
