# Supplementary material for: Genome wide association study of plant height and tiller number in hulless barley
Source: PLoS One. 2021 Dec 2;16(12):e0260723. doi: 10.1371/journal.pone.0260723 (PMC8639095; doi:10.1371/journal.pone.0260723)
Supplement: S3 Fig — (PDF) [file pone.0260723.s003.pdf]

REF	CCC	GGC	GCA	AAT	GCT	ACT	CCT	CAT	TTG	CTA	GAA
	P	G	A	N	A	T	P	H	L	L	E
ALT	CCC	GGC	GCA	AAT	GCT	GCT	CCT	CAT	TTG	CTA	GAA
	P	G	A	N	A	A	P	H	L	L	E
